# Validation of a two‐step approach combining serum biomarkers and liver stiffness measurement to predict advanced fibrosis

**DOI:** 10.1002/jgh3.12590

**Published:** 2021-06-10

**Authors:** Hideki Fujii, Masaru Enomoto, Shinya Fukumoto, Tatsuo Kimura, Yuji Nadatani, Shingo Takashima, Atsushi Hagihara, Sawako Uchida‐Kobayashi, Akihiro Tamori, Naoki Nishimoto, Norifumi Kawada

**Affiliations:** ^1^ Department of Premier Preventive Medicine Graduate School of Medicine, Osaka City University Osaka Japan; ^2^ Department of Hepatology Graduate School of Medicine, Osaka City University Osaka Japan; ^3^ Division of Data Management, Division of Biostatistics Clinical Research and Medical Innovation Center, Hokkaido University Hospital Sapporo Japan

**Keywords:** hepatic fibrosis, non‐alcoholic fatty liver disease, noninvasive fibrosis test, transient elastography

## Abstract

**Background and Aim:**

The Gut and Obesity in Asia Workgroup recently reported that a two‐step approach using fibrosis scores followed by liver stiffness measurement (LSM) could accurately detect patients with non‐alcoholic fatty liver disease (NAFLD) having advanced fibrosis in low‐risk fibrosis populations. This study aimed to validate the utility of this approach using a Japanese health checkup registry.

**Methods:**

This cross‐sectional study included subjects who underwent a health checkup from 2014 to 2019. Using estimated fibrosis stage measured by LSM as a standard, we calculated the percentage of misclassification from assessments made based on fibrosis scores (NAFLD fibrosis score [NFS] or Fibrosis‐4 score [FIB‐4]) and LSM, alone or in combination.

**Results:**

Of 630 subjects with NAFLD, 4 (0.8%) had advanced fibrosis. In the first‐step evaluation, only 21.4–38.0% of subjects needed further testing. This approach was associated with a high specificity of approximately 100% and a negative predictive value of 99.7%. The percentage of misclassification based on NFS or FIB‐4 values followed by LSM in all subjects and using LSM after NFS or FIB‐4 determination only in subjects with indeterminate/high NFS or FIB‐4 values (two‐step approach) was 0% and 0.3% and 0.16% and 0.3%, respectively. In addition, very few false negatives occurred for both NFS and FIB‐4.

**Conclusion:**

The two‐step approach helps to identify the subjects with NAFLD who have advanced fibrosis during a routine health checkup and is associated with only a few false negatives.

## Graphical abstract

We validated “two step approach” using health checkup subjects with nonalcoholic fatty liver disease. In the first‐step evaluation, only 21.4–38.0% of subjects needed further testing. The percentage of misclassification based on NAFLD fibrosis score (NFS) or Fibrosis‐4 score (FIB‐4) values followed by liver stiffness measurement (LSM) in all subjects and using LSM after NFS or FIB‐4 determination only in subjects with indeterminate/high NFS or FIB‐4 values (two‐step approach) was 0% and 0.3% and 0.16% and 0.3%, respectively.

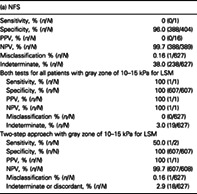

## Introduction

Non‐alcoholic fatty liver disease (NAFLD) is a major form of chronic liver disease with an estimated global prevalence of 25%.^1^ It is a clinical consequence of obesity and can progress to non‐alcoholic steatohepatitis (NASH). NASH is characterized by the presence of steatosis, inflammation, and fibrosis, which ultimately lead to cirrhosis, hepatocellular carcinoma (HCC), or end‐stage liver disease.^2^, ^3^, ^4^

Although the severity of hepatic fibrosis is a key determinant of liver‐related outcomes in NAFLD,^5^, ^6^, ^7^ identifying patients with advanced fibrosis who might benefit from early specialist intervention is challenging.^8^ Screening studies in people at risk of developing NAFLD have shown the prevalence of advanced fibrosis to be 5%, which underlines the need for robust pathways for risk stratification in primary care, with subsequent referrals as required.^9^

Even now, liver biopsy remains the gold standard for diagnosing NASH. However, liver biopsy is compromised by sampling variability and periprocedural risks, such as patient discomfort and rare but severe complications including death.^10^, ^11^ Biopsy costs also make it unsuitable for mass screening, staging, and risk stratification. Noninvasive fibrosis tests (NITs) overcome many limitations of liver biopsy and is now routinely incorporated into specialist clinical practice.^12^, ^13^, ^14^ Simple serum‐based tests (e.g., NAFLD fibrosis score [NFS] and Fibrosis‐4 score [FIB‐4]) involve the measurement of readily available biochemical surrogates and determination of the clinical risk factors for liver fibrosis (e.g., age and sex), respectively. However, when using them, a significant proportion of patients (approximately 30%) fall into the intermediate‐risk category.^14^ Conversely, liver stiffness measurement (LSM) using vibration‐controlled transient elastography (VCTE; FibroScan®, Echosens, Paris, France) is a noninvasive method for determining fibrosis stage, particularly useful for the diagnosis of advanced fibrosis and cirrhosis.^15^ Although LSM is recommended in the current guidelines on the management of NAFLD,^2^, ^3^ it is not widely available because it is expensive and is only available at some expert referral centers. Primary care physicians have been interested in whether a single NIT can identify patients who need further tests, such as liver biopsy. However, considering the cost–benefit balance, it is difficult to make significant discrimination with only a single NIT.^9^

**Figure 1:**
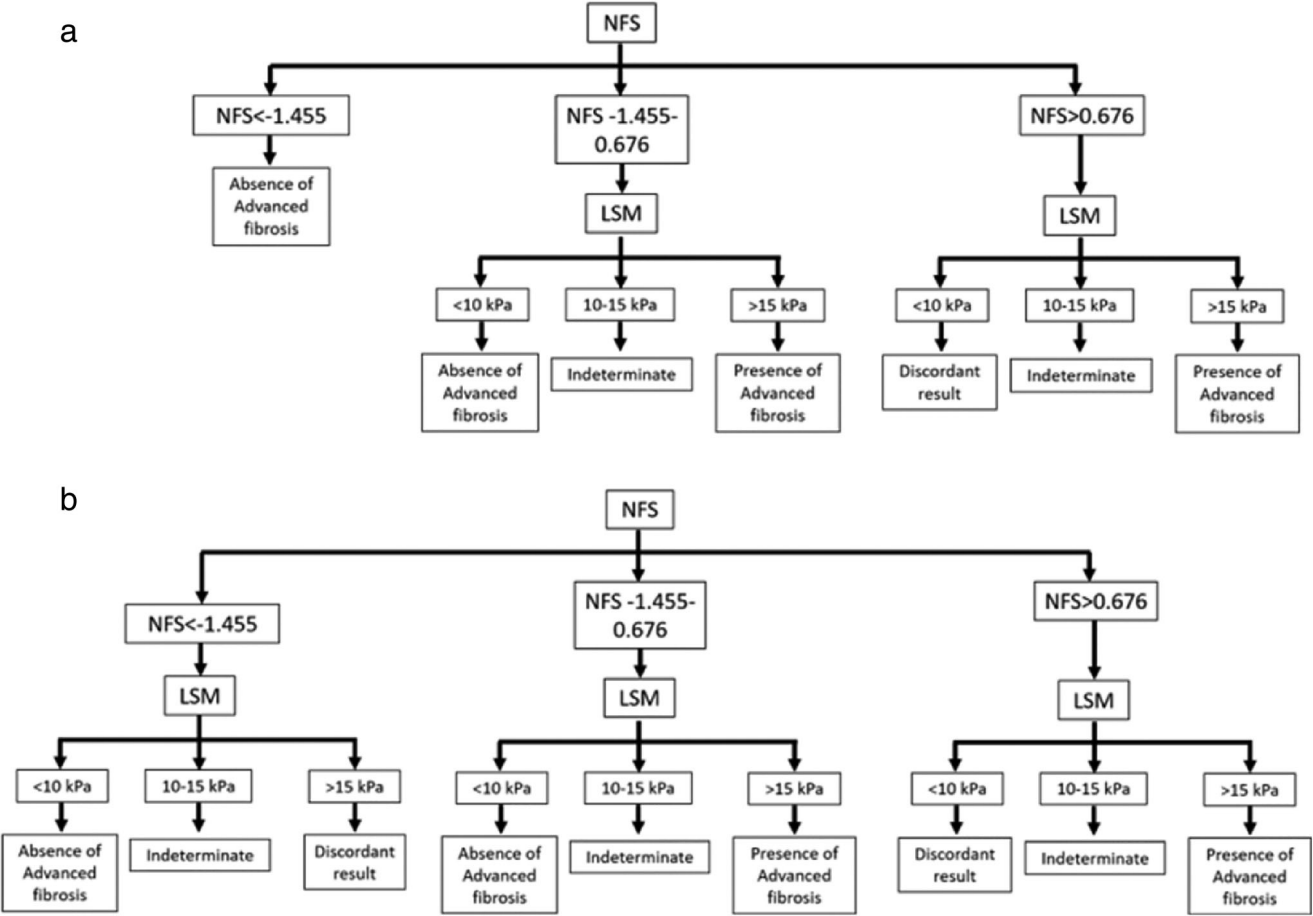
(a) The two‐step approach and (b) both tests for all subjects. LSM, liver stiffness measurement; NAFLD, nonalcoholic liver disease; NFS, NAFLD fibrosis score.

In 2015, Chan et al. developed a two‐step approach.^16^ This approach involves using NFS followed by LSM, but only for patients with indeterminate/high NFS (Fig. 1a). Using this method, selecting only patients with indeterminate and high NFS for LSM facilitated a reduced need for liver biopsy while maintaining the accuracy of diagnosing advanced fibrosis.^16^ In 2019, the Gut and Obesity Asia Workgroup validated this strategy and concluded that using NFS or FIB‐4 followed by LSM for patients with indeterminate/high NFS or FIB‐4 appeared to be the optimal noninvasive approach for the assessment of NAFLD in the general population.^17^ However, the health checkup registry has not been used to verify the use of the two‐step approach.

Thus, we conducted this cross‐sectional study to verify the validity of the two‐step approach for diagnosing advanced fibrosis in patients with NAFLD using a Japanese health checkup registry.

## Methods

### Study population

Subjects were retrospectively selected for this cross‐sectional study from the ongoing MedCity21 health examination registry between April 1, 2014, and September 30, 2019.^18^ We provided an opt‐out option, as explained in the instructions posted on the hospital's website.

**Figure 2:**
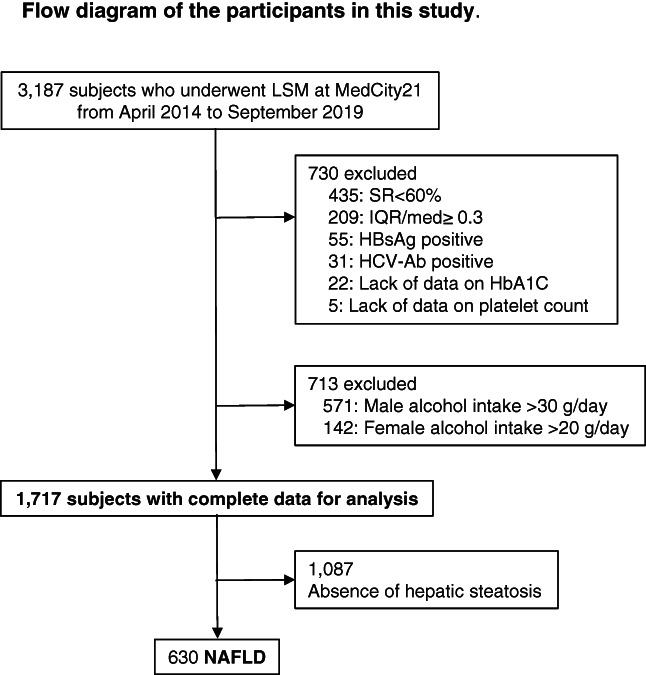
Flow diagram of the study subjects. Ab, antibody; HbA1c, hemoglobin A1c; HBsAg, hepatitis B surface antigen; HBV, hepatitis B virus; HCV, hepatitis C virus; LSM, liver stiffness measurement; SR, success rate; IQR/med, interquartile range/median.

This study included subjects who underwent medical examination, including abdominal ultrasonography and LSM, for the first time within the study period (*n* = 3187). The exclusion criteria were alcohol intake of ≥30 g/day for men (*n* = 571) and ≥20 g/day for women (*n* = 142); success rate (SR) of <60% (*n* = 435); interquartile range/median (IQR/med) for an LSM value of >30% (*n* = 209); positive serology for hepatitis B surface antigen (HBsAg) (*n* = 55); positive serology for hepatitis C virus (HCV) antibodies (*n* = 31); lack of data on hemoglobin A1c (*n* = 22); and lack of data on platelet count (*n* = 5). In total, the data of 1717 subjects were initially analyzed (Fig. 2).

### Clinical assessment

All study subjects underwent a comprehensive health assessment, including medical history, physical examination, laboratory testing, and abdominal ultrasound. Information on smoking habit, medical history, and current drug history was extracted from a standardized questionnaire filled in by the subjects. Anthropometric measurements, including body weight and height, were collected. Body mass index (BMI) was calculated as weight in kilograms, divided by height in meters squared. Waist circumference was measured with a non‐stretchable tape at the level of the umbilicus in the standing position during late expiration. After overnight fasting, blood samples were collected and analyzed following standard laboratory procedures for complete blood count and aspartate aminotransferase (AST), alanine aminotransferase (ALT), and albumin levels. The Lumipulse HBsAg and HCV assay (Fujirebio Inc., Tokyo, Japan) was used to measure the levels of serum markers, including HBsAg and anti‐HCV antibodies.

Subjects who had a fasting blood glucose level of ≥126 mg/dL or hemoglobin A1c level of ≥6.5% or those undergoing treatment for diabetes were defined as subjects with diabetes mellitus (DM). Similarly, a systolic blood pressure of ≥140 mmHg, diastolic blood pressure of ≥90 mmHg, or ongoing treatment for hypertension were defined as hypertension. A serum total cholesterol level of ≥220 mg/dL, high‐density lipoprotein cholesterol level of <40 mg/dL, and/or triglyceride level of ≥150 mg/dL or ongoing treatment for dyslipidemia were defined as dyslipidemia.

### Alcohol intake‐specific screening measures and definition of NAFLD

The frequency of alcohol intake was assessed according to the volume consumed. Daily alcohol consumption was calculated in grams using a modified template from our previous research.^19^ Daily alcohol consumption (g/day) was calculated as follows: (frequency of alcohol intake) × (average alcohol consumption [g])/7.

Diagnosis of NAFLD was based on ultrasound evidence of fatty liver disease and the exclusion of both secondary causes such as viral hepatitis and excessive alcohol consumption (≥30 g/day for males and ≥20 g/day for females).^2^, ^3^

### Abdominal ultrasonography and assessment of disease severity

Fatty liver was diagnosed by abdominal ultrasonography using the Toshiba Aplio 500 device (Toshiba Medical Systems Corporation, Ohtawara, Japan). Abdominal ultrasonography was performed at MedCity21 by experienced medical sonographers registered with the Japan Society of Ultrasonics in Medicine. Hepatic steatosis was semi‐quantified according to the criteria described by Hamaguchi.^20^

The severity of liver fibrosis was assessed using two noninvasive markers in subjects with fatty liver. NFS was calculated using the following formula: −1.675 + 0.037 × age (years) + 0.094 × BMI (kg/m^2^) + 1.13 × impaired glucose tolerance/DM (yes = 1, no = 0) + 0.99 × AST to ALT ratio − 0.013 × platelet (×10^9^/L) − 0.66 × albumin (g/dL).^21^ FIB‐4 was calculated as follows: age (years) × AST (U/L)/(platelet count [×10^9^/L] × ALT [U/L]^1/2^).^22^

### Vibration‐controlled transient elastography

VCTE was performed using an M‐probe device. Details of the technique and examination procedure for LSM have been described previously.^23^ Diagnosis of advanced fibrosis was according to the report by Chan et al.^17^ An LSM value of <10 kPa indicated the absence of advanced fibrosis and ≥15 kPa indicated the presence of advanced fibrosis. An LSM value between 10 and 14.9 kPa was considered indeterminate. The controlled attenuation parameter (CAP) was also measured using VCTE to stage steatosis. CAP was calculated using a proprietary algorithm based on the ultrasonic attenuation coefficient of the shear wave of VCTE, an estimate of the total ultrasonic attenuation at 3.5 MHz. Only VCTE measurements based on at least 10 valid shots, SR of ≥60%, and IQR/med of <30% were considered reliable and used for statistical analysis.

### Definition of two‐step approach‐related terms and modification in this study

We defined the two‐step approach‐related terms used in this study as follows.

NFS alone: Diagnosed as a low/indeterminant/high risk of developing advanced fibrosis only by NFS. A score below −1.455 indicated the absence of advanced fibrosis, and a score above 0.676 indicated the presence of advanced fibrosis. A score of −1.455 to 0.676 was considered indeterminate.^21^

FIB‐4 alone: Diagnosed as a low/indeterminant/high risk of developing advanced fibrosis only based on FIB‐4. A score of <1.45 indicated the absence of advanced fibrosis, and a score of >3.25 indicated the presence of advanced fibrosis. A score between 1.45 and 3.25 was considered indeterminate.^17^, ^24^

Both tests: Serum‐based NITs (FIB‐4 or NFS) and LSM (Fig. 1b).

Discordant results: Results showing high risk by FIB‐4 or NFS but an LSM value of <10 kPa.

Misclassification: The sum of a false‐positive and false‐negative results in the diagnosis of advanced fibrosis.

We made some modifications to the methods reported by Chan et al.^16^, ^17^ By the assessment of LSM obtained using VCTE, we estimated the fibrosis stage using cutoff values that have been validated for Japanese patients with biopsy‐proven NAFLD^25^ as follows: F0, LSM <5.9 kPa; F1, LSM = 5.9 to <6.7 kPa; F2, LSM = 6.7 to <9.8 kPa; F3, LSM = 9.8 to <17.5 kPa; and F4, LSM ≥17.5 kPa (Table 1). F3 and F4 were defined as advanced fibrosis.

**Table 1:** The number and proportion of the patients with each fibrosis stage estimated by VCTE

Fibrosis stage estimated by VCTE	NAFLD (*n* = 630)^‡^
F0 (LSM <5.9 kPa)^†^	549 (87.1)
F1 (5.9 ≤ LSM < 6.7 kPa)^†^	41 (6.5)
F2 (6.7 ≤ LSM < 9.8 kPa)^†^	36 (5.7)
F3 (9.8 ≤ LSM < 17.5 kPa)^†^	2 (0.4)
F4 (17.5 ≤ LSM kPa)^†^	2 (0.4)

^†^ Number (%).

^‡^ Mean (standard deviation). F3 and F4 were defined as advanced fibrosis.

LSM, liver stiffness measurement; NAFLD, non‐alcoholic fatty liver disease; VCTE, vibration controlled transient elastography.

### Statistical analysis

Subject characteristics at baseline were compared using the chi‐square test and t‐test for categorical and continuous variables, respectively. To calculate diagnostic test characteristics, a 2‐by‐2 table was constructed with the presence/absence of advanced fibrosis according to LSM reading, against the presence/absence of advanced fibrosis according to noninvasive fibrosis scores using the established thresholds. Sensitivity, specificity, positive predictive value (PPV), and negative predictive value (NPV) were calculated. The performance of NFS and FIB‐4 for the diagnosis of advanced fibrosis was determined using area under the receiver operating characteristic curve (AUROC). A *P* value of <0.05 was considered to indicate statistical significance. Statistical analyses were conducted using JMP 13.0.0 (SAS Institute Inc., Cary, NC, USA).

## Results

### Characteristics of the study population

**Figure 3:**
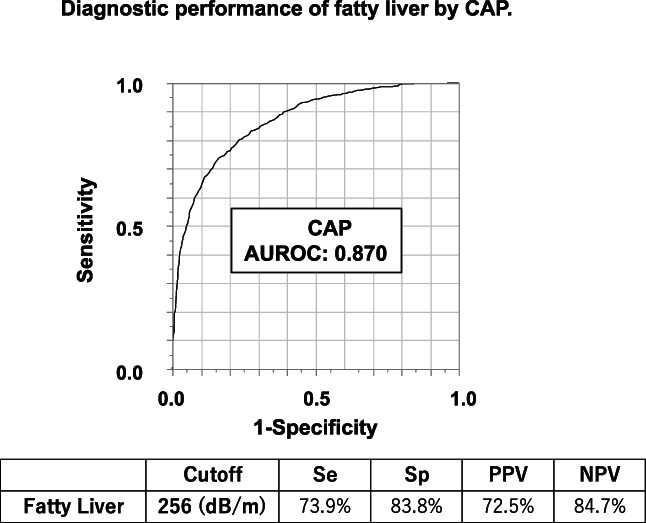
Diagnostic performance of fatty liver based on CAP. AUROC, area under the receiver operating characteristic curve; CAP, controlled attenuation parameter; NPV, negative predictive value; PPV, positive predictive value; Se, sensitivity; Sp, specificity.

The study population comprised 1717 subjects who met the inclusion criteria (Fig. 2). Of these, 1114 subjects were excluded because hepatic steatosis was not detected on ultrasound. Thus, 630 subjects were diagnosed with NAFLD. The characteristics of the subjects are summarized in Table 2. Figure 3 shows diagnostic performance of fatty liver by CAP (*n* = 1717). The AUROC of fatty liver was 0.870, and the optimal cutoff was 256 dB/m.

**Table 2:** Clinical characteristics of the patients

Variables	NAFLD (*n* = 630)
Age (years)^†^	53 (46–63)
Male, *n* (%)^‡^	437 (69)
BMI (kg/m^2^)^†^	25.2 (23.4–26.9)
Waist circumference (cm)^†^	90.5 (86–95)
T2DM^‡^	70 (11)
Hypertension^‡^	171 (27)
Dyslipidemia^‡^	147 (23)
Platelet count (10^9^/mL)^†^	231 (196–262)
AST (U/L)^†^	22 (19–28)
ALT (U/L)^†^	26 (19–40)
FIB‐4^†^	1.03 (0.74–1.36)
NFS^†^	−1.88 (−2.81–0.99)
VCTE	
LSM (kPa)^†^	4.1 (3.5–4.8)
CAP (dB/m)^†^	287 (253–316)

^†^ Median (interquartile range).

^‡^ Number (%).

ALT, alanine transaminase; AST, aspartate aminotransferase; CAP, controlled attenuation parameter; LSM, liver stiffness measurement; NAFLD, non‐alcoholic fatty liver disease; VCTE, vibration controlled transient elastography.

**Figure 4:**
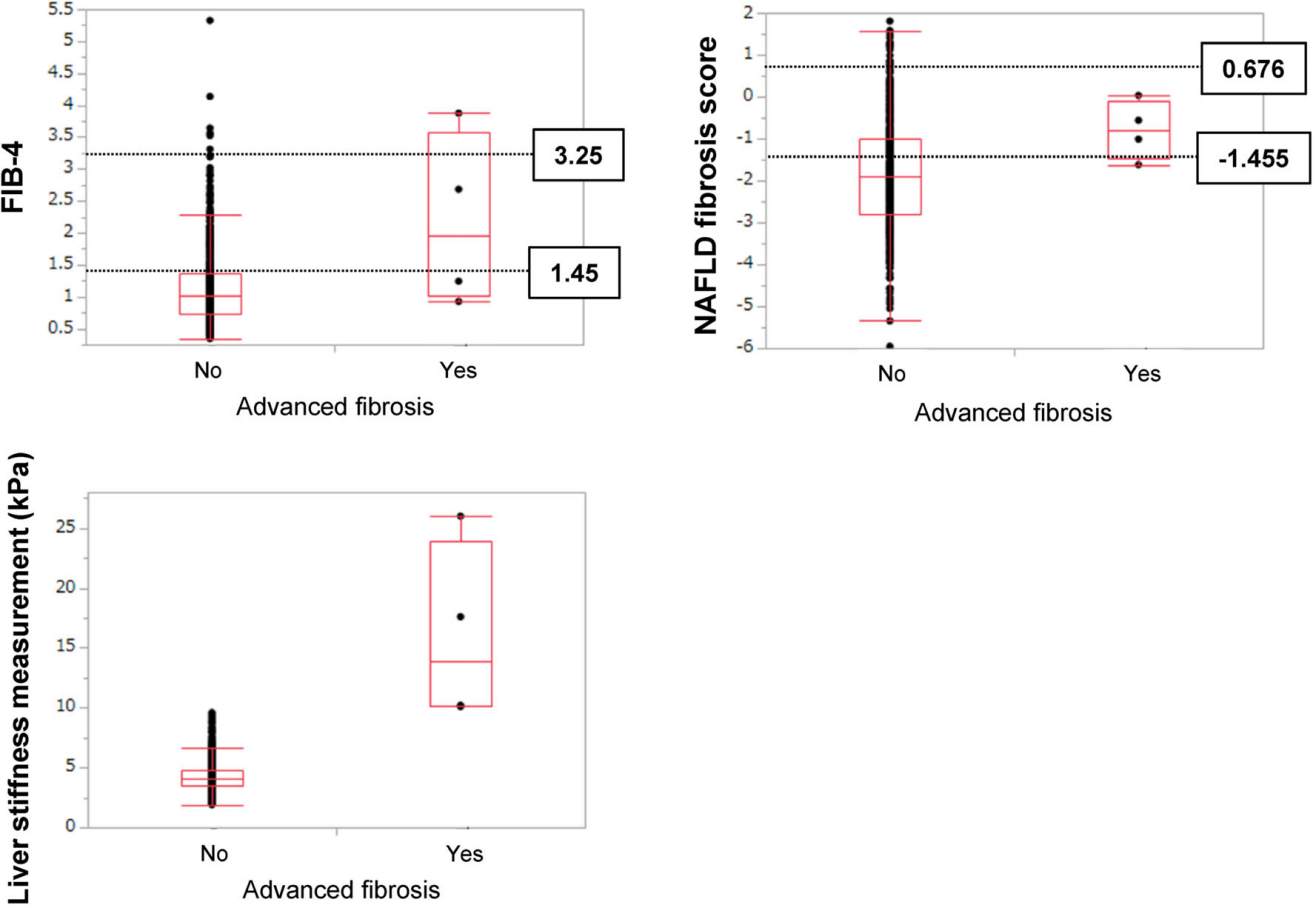
Boxplots showing the NAFLD fibrosis score, FIB‐4 value, and liver stiffness measurement value of subjects with and without advanced fibrosis in the overall study population. FIB‐4, Fibrosis‐4; NAFLD, non‐alcoholic fatty liver disease.

The number and proportion of subjects with each fibrosis stage, as estimated by VCTE, are shown in Table 1. Four subjects (0.8%) were diagnosed with advanced fibrosis. Figure 4 shows boxplots representing NFS and FIB‐4 for subjects with and without advanced fibrosis in the overall study population. Compared with subjects without advanced fibrosis, those with advanced fibrosis were more likely to have higher FIB‐4 values (median 1.14 *vs* 2.18, *P* = 0.0005). However, NFS did not reach significant differences (−1.87 *vs* −0.79, *P* = 0.0837).

### Combination of NFS and LSM for diagnosis of advanced fibrosis

The data for albumin were missing in three subjects. Therefore, a total of 627 subjects were finally analyzed. Using the two‐step approach, the next LSM could be omitted in 62.0% (*n* = 389) of the subjects (Figure S1). Table 3, part a shows the sensitivity, specificity, PPV, NPV, percentage of misclassification, and indeterminate/discordant results using NFS and other approaches. Regarding NFS alone and the two‐step approach, sensitivity increased from 0 to 50%, specificity increased from 96 to 100%, PPV increased from 0 to 100%, and NPV remained unchanged at 99.7%. The corresponding cross‐tabulations and additional notes can be found in Figures S1 and S2.

**Table 3:** Sensitivity, specificity, positive predictive value, negative predictive value, percentage of misclassification, and indeterminate or discordant results for (a) NFS and (b) FIB‐4

(a) NFS	
Sensitivity, % (*n*/*N*)	0 (0/1)
Specificity, % (*n*/*N*)	96.0 (388/404)
PPV, % (*n*/*N*)	0 (0/16)
NPV, % (*n*/*N*)	99.7 (388/389)
Misclassification % (*n*/*N*)	0.16 (1/627)
Indeterminate, % (*n*/*N*)	38.0 (238/627)
Both tests for all patients with gray zone of 10–15 kPa for LSM	
Sensitivity, % (*n*/*N*)	100 (1/1)
Specificity, % (*n/N*)	100 (607/607)
PPV, % (*n/N*)	100 (1/1)
NPV, % (*n/N*)	100 (1/1)
Misclassification % (*n/N*)	0 (0/627)
Indeterminate, % (*n/N*)	3.0 (19/627)
Two‐step approach with gray zone of 10–15 kPa for LSM	
Sensitivity, % (*n/N*)	50.0 (1/2)
Specificity, % (n/N)	100 (607/607)
PPV, % (*n*/*N*)	100 (1/1)
NPV, % (*n*/*N*)	99.7 (607/608)
Misclassification % (*n*/*N*)	0.16 (1/627)
Indeterminate or discordant, % (*n*/*N*)	2.9 (18/627)

(b) FIB‐4	
Sensitivity, % (*n*/*N*)	33.3 (1/3)
Specificity, % (*n*/*N*)	98.8 (493/499)
PPV, % (*n*/*N*)	14.3 (1/7)
NPV, % (*n*/*N*)	99.6 (493/495)
Misclassification % (*n*/*N*)	0 (0/630)
Indeterminate, % (*n*/*N*)	20.3 (128/630)
Both tests for all patients with gray zone of 10–15 kPa for LSM	
Sensitivity, % (*n*/*N*)	50.0 (2/4)
Specificity, % (*n*/*N*)	100 (620/620)
PPV, % (*n*/*N*)	50.0 (2/4)
NPV, % (*n*/*N*)	99.7 (620/622)
Misclassification % (*n*/*N*)	0.3 (2/630)
Indeterminate, % (*n*/*N*)	1.0 (6/630)
Two‐step approach with gray zone of 10–15 kPa for LSM	
Sensitivity, % (*n*/*N*)	50.0 (2/4)
Specificity, % (*n*/*N*)	100 (620/620)
PPV, % (*n*/*N*)	100 (2/2)
NPV, % (*n*/*N*)	99.7 (607/608)
Misclassification % (*n*/*N*)	0.3 (2/630)
Indeterminate or discordant, % (*n*/*N*)	0.95 (6/630)

Data are presented as number (%).

Abbreviations: LSM, liver stiffness measurement; FIB‐4, Fibrosis‐4; NFS, NAFLD fibrosis score; NPV, negative predictive value; PPV, positive predictive value.

### Combination of FIB‐4 and LSM for diagnosis of advanced fibrosis

Using the two‐step approach, the next LSM could be omitted in 78.6% (*n* = 495) of subjects (Figure S3). Table 3, part b shows the sensitivity, specificity, PPV, NPV, percentage of misclassification, and indeterminate/discordant results using FIB‐4 and other approaches. Regarding FIB‐4 alone and the two‐step approach, sensitivity increased from 33.3 to 50%, specificity increased from 98.8 to 100%, PPV increased from 14.3 to 100%, and NPV increased from 99.6 to 99.7%. The corresponding cross‐tabulations and additional notes can be found in Figures S3 and S4.

### Case presentation

Table S1 shows the clinical characteristics of the subjects with advanced fibrosis. In Case 1 and Case 2, FIB‐4 alone and the two‐step approach using FIB‐4 misdiagnosed advanced fibrosis. In Case 3, NFS alone and the two‐step approach using NFS misdiagnosed advanced fibrosis. In Case 4, none of the methods misdiagnosed advanced fibrosis.

### Diagnostic ability of NFS and FIB‐4 for advanced fibrosis

The AUROC of NFS and FIB‐4 for diagnosing advanced fibrosis across the study population is shown in Table S2. Moreover, as reported by Chan et al.,^16^ the AUROC of FIB‐4 was numerically higher than that of NFS.

## Discussion

We validated the utility of the two‐step approach using the Japanese health checkup registry. Using this two‐step approach, only 21.4–38.0% of subjects required further evaluation with transient elastography (TE). The results using the two‐step approach were associated with specificity close to 100% and NPV of 99.7%. Besides, there were a few false negatives in both FIB‐4 and NFS, indicating that people without advanced fibrosis can be confidently excluded but with a minimal possibility of a missed diagnosis. In addition, unlike the report by Chan et al.,^16^ using both tests for all patients can potentially result in a low percentage of discordant results.

The vast majority of referrals made to secondary care hepatologists could have been managed in primary care. Reducing inappropriate referrals represents an opportunity to reduce unnecessary investigations, inconvenience, and even harm to patients; pressure on secondary care services; and cost borne by the healthcare system.^8^ Simultaneously, it is important to provide the appropriate opportunity to patients who need a referral. Therefore, previous studies have recommended a combination of NITs: (i) enhanced liver fibrosis score (ELF™; Siemens Healthcare, Erlangen, Germany)^26^ and LSM^27^; (ii) NFS and LSM^16^, ^28^; (iii) FIB‐4 and ELF™,^8^ and (iv) NFS/FIB‐4 and LSM.^17^ Although each combination has shown high specificity (>95%) and NPV (85–95%), false‐negative rates of 5–15% were also observed.^16^, ^17^, ^27^, ^28^ Interestingly, Chan et al. reported that a two‐step approach was better than using both tests for all patients because the two‐step approach produced a lower rate of indeterminate/discordant results than both tests for all patients (27.5% *vs* 10.1%).^17^ However, using the two‐step approach can result in missing out patients with a low risk diagnosed in the first step (NFS or FIB‐4), who may have indeterminate/discordant results when both tests are used. Moreover, the percentage of indeterminate/discordant results increased with a higher percentage for the diagnosis of advanced fibrosis in all patients. Chan et al. reported that a total of 24% of patients had advanced fibrosis.

Assuming the prevalence of advanced fibrosis to be <5% in unselected patients with NAFLD, the NPV of these tests is >98%.^29^ For example, Mahady et al. reported that in 749 general ambulatory individuals, 15 had advanced fibrosis (2%).^30^ Lee et al. reported that of 1178 TE‐defined NAFLD patients who underwent a medical health checkup, 1.8% had advanced fibrosis (LSM ≥9.5 kPa).^31^ Chan et al. investigated a subpopulation with 3.7% cases of advanced fibrosis.^17^ They reported that the percentage of indeterminate/discordant results using NFS alone, both tests for all patients, and the two‐step approach using LSM only for patients with indeterminate and high NFS values was 24.6%, 19.2%, and 6.9%, respectively. Moreover, our data showed that the rate of misclassification was 2.2%, 1.7%, and 2.7%, respectively. Therefore, we concluded that the two‐step approach is a useful method with high specificity and NPV for diagnosing health checkup subjects with a low rate of diagnosis of advanced fibrosis. Furthermore, a false‐negative rate of <1% was observed.

All except one subject with a low NFS value had an LSM value of ≥10 kPa, whereas all except two subjects with a low FIB‐4 value had an LSM value of ≥10 kPa. These findings support the hypothesis that using the two‐step approach only for patients with indeterminate/high NFS or FIB‐4 values will require LSM, resulting in a more efficient use of healthcare resources.

In the simulated general population with a prevalence of advanced fibrosis of 3.7% in the study by Chan et al.,^16^ only 3 of 469 (0.6%) patients with an LSM value of <10 kPa had histological advanced fibrosis. Therefore, patients with low NFS or FIB‐4 values in this study likely did not have histological advanced fibrosis. Conversely, only 19 of 124 (15.3%) subjects with an LSM value of ≥10 kPa had histologically advanced fibrosis. This indicates that the four subjects identified as having advanced fibrosis by LSM in this study possibly did not have histologically advanced fibrosis, particularly the two subjects with an LSM value of slightly above 10 kPa. However, liver biopsy is also limited by sampling and observer variability.^11^, ^32^

Several limitations must be considered while interpreting the study results. First, the main limitation of this study was the use of LSM as a reference standard. Therefore, we cannot accurately determine whether the patients have histologically advanced fibrosis. Second, this was a retrospective single‐center study. Third, selection bias was a major limitation. Most subjects were healthy enough to engage in work and were sufficiently conscious of their health to voluntarily undergo a health checkup.^33^ Therefore, the study results may not apply to individuals who are not generally healthy. Fourth, individuals who include LSM in the annual health checkup tend to be those who can afford it, which might be one of the causes of selection bias. Fifth, when using LSM, technical failure was a common phenomenon ranging in the frequency of 6.7–27.0%, and this was primarily related to a high BMI.^15^, ^34^ In our analysis, 20.2% of subjects showed unreliable measurements. For such patients, it may be necessary to consider the two‐step approach with two serum‐derived NITs. Lastly, the proportion of advanced fibrosis was low (0.4%). However, our data are composed of real‐world data from a health checkup; therefore, it is expected that our research will be useful for diagnosing advanced liver fibrosis during health checkups in a generally healthy population.

In conclusion, we validated a two‐step approach using a Japanese health checkup registry. This approach was associated with a very high specificity of approximately 100% and an NPV of 99.7%. Following the first‐step evaluation, only 21.4–38.0% of subjects would need further testing. The two‐step approach is extremely useful for selecting patients with NAFLD who have advanced fibrosis in health examinations with minimal false negatives.

## Ethics approval statement

The MedCity21 health examination registry protocol was a comprehensive agreement approved by the Ethics Committee (approval no. 2927). This study on liver disease was part of the MedCity21 health examination registry and conducted in full accordance with the tenets of the Declaration of Helsinki, and the study protocol was approved by the Ethics Committee (approval no. 2019‐076, February 21, 2020).

## Supporting information


**Figure S1.** Two‐step approach with a gray zone of 10 to 15 kPa for liver stiffness measurement for the prediction of advanced fibrosis in the overall population.
**Figure S2**. Both test (NAFLD fibrosis score and liver stiffness measurement) for all patients with a gray zone of 10 to 15 kPa for liver stiffness measurement for the prediction of advanced fibrosis in the overall population.
**Figure S3**. Two‐step approach with a gray zone of 10 to 15 kPa for liver stiffness measurement for the prediction of advanced fibrosis in the overall population.
**Figure S4**. Both test (FIB‐4 and liver stiffness measurement) for all patients with a gray zone of 10 to 15 kPa for liver stiffness measurement for the prediction of advanced fibrosis in the overall population.
**Table S1**. Clinical characteristics of the subjects with advanced fibrosis.
**Table S2**. The AUROC (95% CI) of the NFS and the FIB‐4 for the diagnosis of advanced fibrosis.

## References

[1] Younossi ZM , Koenig AB , Abdelatif D , Fazel Y , Henry L , Wymer M . Global epidemiology of nonalcoholic fatty liver disease‐meta‐analytic assessment of prevalence, incidence, and outcomes. Hepatology. 2016; 64: 73–84. 26707365 10.1002/hep.28431

[2] Chalasani N , Younossi Z , Lavine JE *et al*. The diagnosis and management of nonalcoholic fatty liver disease: practice guidance from the American Association for the Study of Liver Diseases. Hepatology. 2018; 67: 328–57. 28714183 10.1002/hep.29367

[3] European Association for the Study of the Liver (EASL), European Association for the Study of Diabetes (EASD); European Association for the Study of Obesity (EASO) . EASL‐EASD‐EASO Clinical Practice Guidelines for the management of non‐alcoholic fatty liver disease. J. Hepatol. 2016; 64: 1388–402. 27062661 10.1016/j.jhep.2015.11.004

[4] Wong VW , Chan WK , Chitturi S *et al*. Asia‐Pacific Working Party on Non‐alcoholic Fatty Liver Disease guidelines 2017‐Part 1: Definition, risk factors and assessment. J. Gastroenterol. Hepatol. 2018; 33: 70–85. 28670712 10.1111/jgh.13857

[5] Angulo P , Kleiner DE , Dam‐Larsen S *et al*. Liver fibrosis, but no other histologic features, is associated with long‐term outcomes of patients with nonalcoholic fatty liver disease. Gastroenterology. 2015; 149: 389–97. 25935633 10.1053/j.gastro.2015.04.043 PMC4516664

[6] Dulai PS , Singh S , Patel J *et al*. Increased risk of mortality by fibrosis stage in nonalcoholic fatty liver disease: systematic review and meta‐analysis. Hepatology. 2017; 65: 1557–65. 28130788 10.1002/hep.29085 PMC5397356

[7] Estes C , Anstee QM , Arias‐Loste MT *et al*. Modeling NAFLD disease burden in China, France, Germany, Italy, Japan, Spain, United Kingdom, and United States for the period 2016‐2030. J. Hepatol. 2018; 69: 896–904. 29886156 10.1016/j.jhep.2018.05.036

[8] Srivastava A , Gailer R , Tanwar S *et al*. Prospective evaluation of a primary care referral pathway for patients with non‐alcoholic fatty liver disease. J. Hepatol. 2019; 71: 371–8. 30965069 10.1016/j.jhep.2019.03.033

[9] Tsochatzis EA , Newsome PN . Non‐alcoholic fatty liver disease and the interface between primary and secondary care. Lancet Gastroenterol. Hepatol. 2018; 3: 509–17. 29893235 10.1016/S2468-1253(18)30077-3

[10] Bravo AA , Sheth SG , Chopra S . Liver biopsy. N. Engl. J. Med. 2001; 344: 495–500. 11172192 10.1056/NEJM200102153440706

[11] Ratziu V , Charlotte F , Heurtier A *et al*. Sampling variability of liver biopsy in nonalcoholic fatty liver disease. Gastroenterology. 2005; 128: 1898–906. 15940625 10.1053/j.gastro.2005.03.084

[12] Castera L . Non‐invasive tests for liver fibrosis in NAFLD: Creating pathways between primary healthcare and liver clinics. Liver Int. 2020; 40: 77–81. 32077617 10.1111/liv.14347

[13] Loomba R , Adams LA . Advances in non‐invasive assessment of hepatic fibrosis. Gut. 2020; 69: 1343–52. 32066623 10.1136/gutjnl-2018-317593 PMC7945956

[14] Castera L , Friedrich‐Rust M , Loomba R . Noninvasive assessment of liver disease in patients with nonalcoholic fatty liver disease. Gastroenterology. 2019; 156: 1264–81. 30660725 10.1053/j.gastro.2018.12.036 PMC7505052

[15] Wong VW , Vergniol J , Wong GL *et al*. Diagnosis of fibrosis and cirrhosis using liver stiffness measurement in nonalcoholic fatty liver disease. Hepatology. 2010; 51: 454–62. 20101745 10.1002/hep.23312

[16] Chan WK , Nik Mustapha NR , Mahadeva S . A novel 2‐step approach combining the NAFLD fibrosis score and liver stiffness measurement for predicting advanced fibrosis. Hepatol Int. 2015; 9: 594–602. 25788185 10.1007/s12072-014-9596-7

[17] Chan WK , Treeprasertsuk S , Goh GB *et al*. Optimizing use of nonalcoholic fatty liver disease fibrosis score, fibrosis‐4 score, and liver stiffness measurement to identify patients with advanced fibrosis. Clin. Gastroenterol. Hepatol. 2019; 17: 2570–80. 30876959 10.1016/j.cgh.2019.03.006

[18] Yoshida S , Kurajoh M , Fukumoto S *et al*. Association of plasma xanthine oxidoreductase activity with blood pressure affected by oxidative stress level: MedCity21 health examination registry. Sci. Rep. 2020; 10: 4437. 32157204 10.1038/s41598-020-61463-8 PMC7064483

[19] Fujii H , Nishimoto N , Yamaguchi S *et al*. The alcohol use disorders identification test for consumption (AUDIT‐C) is more useful than pre‐existing laboratory tests for predicting hazardous drinking: a cross‐sectional study. BMC Public Health. 2016; 16: 379–86. 27165437 10.1186/s12889-016-3053-6 PMC4862044

[20] Hamaguchi M , Kojima T , Itoh Y *et al*. The severity of ultrasonographic findings in nonalcoholic fatty liver disease reflects the metabolic syndrome and visceral fat accumulation. Am. J. Gastroenterol. 2007; 102: 2708–15. 17894848 10.1111/j.1572-0241.2007.01526.x

[21] Angulo P , Hui JM , Marchesini G *et al*. The NAFLD fibrosis score: a noninvasive system that identifies liver fibrosis in patients with NAFLD. Hepatology. 2007; 45: 846–54. 17393509 10.1002/hep.21496

[22] Sterling RK , Lissen E , Clumeck N *et al*. Development of a simple noninvasive index to predict significant fibrosis in patients with HIV/HCV coinfection. Hepatology. 2006; 43: 1317–25. 16729309 10.1002/hep.21178

[23] Yoneda M , Yoneda M , Mawatari H *et al*. Noninvasive assessment of liver fibrosis by measurement of stiffness in patients with nonalcoholic fatty liver disease (NAFLD). Dig. Liver Dis. 2008; 40: 371–8. 18083083 10.1016/j.dld.2007.10.019

[24] Sumida Y , Yoneda M , Hyogo H *et al*. Validation of the FIB4 index in a Japanese nonalcoholic fatty liver disease population. BMC Gastroenterol. 2012; 12: 2–10. 22221544 10.1186/1471-230X-12-2 PMC3266187

[25] Tomeno W , Imajo K , Kuwada Y *et al*. Distribution of liver stiffness in non‐alcoholic fatty liver disease with higher fibrosis‐4 index than low cut‐off index. J. Gastroenterol. Hepatol. 2019; 34: 1411–146. 30506860 10.1111/jgh.14559

[26] Guha IN , Parkes J , Roderick P *et al*. Noninvasive markers of fibrosis in nonalcoholic fatty liver disease: validating the European Liver Fibrosis Panel and exploring simple markers. Hepatology. 2008; 47: 455–60. 18038452 10.1002/hep.21984

[27] Patel P , Hossain F , Horsfall LU *et al*. A pragmatic approach identifies a high rate of nonalcoholic fatty liver disease with advanced fibrosis in diabetes clinics and at‐risk populations in primary care. Hepatol Commun. 2018; 2: 893–905. 30094401 10.1002/hep4.1208 PMC6078214

[28] Petta S , Vanni E , Bugianesi E *et al*. The combination of liver stiffness measurement and NAFLD fibrosis score improves the noninvasive diagnostic accuracy for severe liver fibrosis in patients with nonalcoholic fatty liver disease. Liver Int. 2015; 35: 1566–73. 24798049 10.1111/liv.12584

[29] Crossan C , Tsochatzis EA , Longworth L *et al*. Cost‐effectiveness of non‐invasive methods for assessment and monitoring of liver fibrosis and cirrhosis in patients with chronic liver disease: systematic review and economic evaluation. Health Technol. Assess. 2015; 19: 1–409. 10.3310/hta19090 PMC4781028 25633908

[30] Mahady SE , Macaskill P , Craig JC *et al*. diagnostic accuracy of noninvasive fibrosis scores in a population of individuals with a low prevalence of fibrosis. Clin. Gastroenterol. Hepatol. 2017; 15: 1453–60. 28286195 10.1016/j.cgh.2017.02.031

[31] Lee HW , Kim BK , Kim SU *et al*. Prevalence and predictors of significant fibrosis among subjects with transient elastography‐defined nonalcoholic fatty liver disease. Dig. Dis. Sci. 2017; 62: 2150–8. 28523578 10.1007/s10620-017-4592-0

[32] Davison BA , Harrison SA , Cotter G *et al*. Suboptimal reliability of liver biopsy evaluation has implications for randomized clinical trials. J. Hepatol. 2020; 73: 1322–32. 32610115 10.1016/j.jhep.2020.06.025

[33] Moriya A , Iwasaki Y , Ohguchi S *et al*. Roles of alcohol consumption in fatty liver: a longitudinal study. J. Hepatol. 2015; 62: 921–7. 25433160 10.1016/j.jhep.2014.11.025

[34] Yoneda M , Honda Y , Nogami A *et al*. Advances in ultrasound elastography for nonalcoholic fatty liver disease. J Med Ultrason. 2001) 2020; 47: 521–33. 10.1007/s10396-020-01040-8 32748075

