# Supplementary material for: Validation of a two‐step approach combining serum biomarkers and liver stiffness measurement to predict advanced fibrosis
Source: JGH Open. 2021 Jun 10;5(7):801–8. doi: 10.1002/jgh3.12590 (PMC8264241; doi:10.1002/jgh3.12590)
Supplement: Supplementary file 1 — Figure S1. Two‐step approach with a gray zone of 10 to 15 kPa for liver stiffness measurement for the prediction of advanced fibrosis in the overall population. Figure S2. Both test (NAFLD fibrosis score and liver stiffness measurement) for all patients with a gray zone of 10 to 15 kPa for liver stiffness measurement for the prediction of advanced fibrosis in the overall population. Figure S3. Two‐step approach with a gray zone of 10 to 15 kPa for liver stiffness measurement for the prediction of advanced fibrosis in the overall population. Figure S4. Both test (FIB‐4 and liver stiffness measurement) for all patients with a gray zone of 10 to 15 kPa for liver stiffness measurement for the prediction of advanced fibrosis in the overall population. Table S1. Clinical characteristics of the subjects with advanced fibrosis. Table S2. The AUROC (95% CI) of the NFS and the FIB‐4 for the diagnosis of advanced fibrosis. [file JGH3-5-801-s001.pptx]

## Slide 1
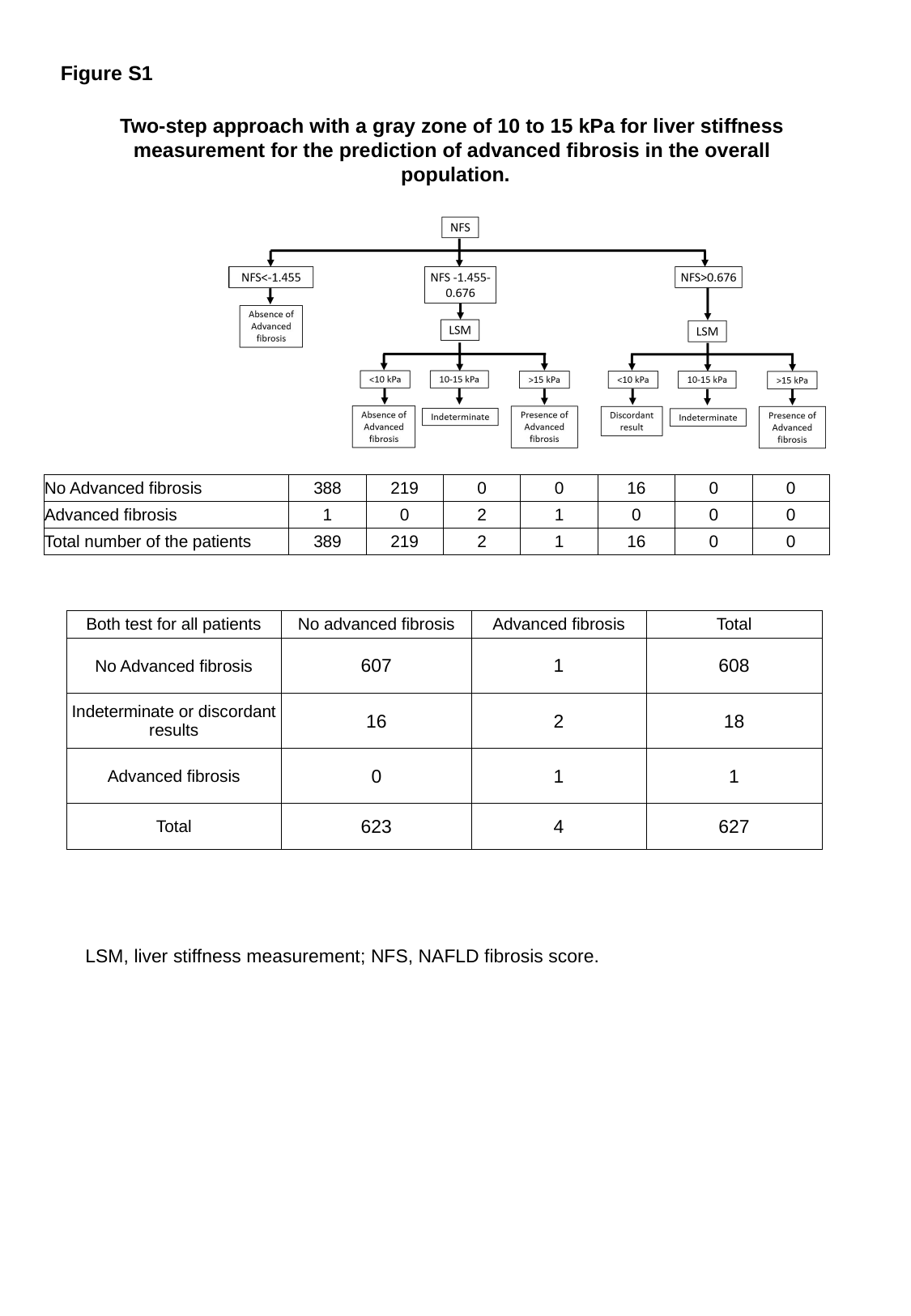

Figure S1
Two-step approach with a gray zone of 10 to 15 kPa for liver stiffness
measurement for the prediction of advanced ﬁbrosis in the overall
population.
| No Advanced fibrosis | 388 | 219 | 0 | 0 | 16 | 0 | 0 |
| --- | --- | --- | --- | --- | --- | --- | --- |
| Advanced fibrosis | 1 | 0 | 2 | 1 | 0 | 0 | 0 |
| Total number of the patients | 389 | 219 | 2 | 1 | 16 | 0 | 0 |
| Both test for all patients | No advanced fibrosis | Advanced fibrosis | Total |
| --- | --- | --- | --- |
| No Advanced fibrosis | 607 | 1 | 608 |
| Indeterminate or discordant results | 16 | 2 | 18 |
| Advanced fibrosis | 0 | 1 | 1 |
| Total | 623 | 4 | 627 |
LSM, liver stiffness measurement; NFS, NAFLD fibrosis score.

## Slide 2
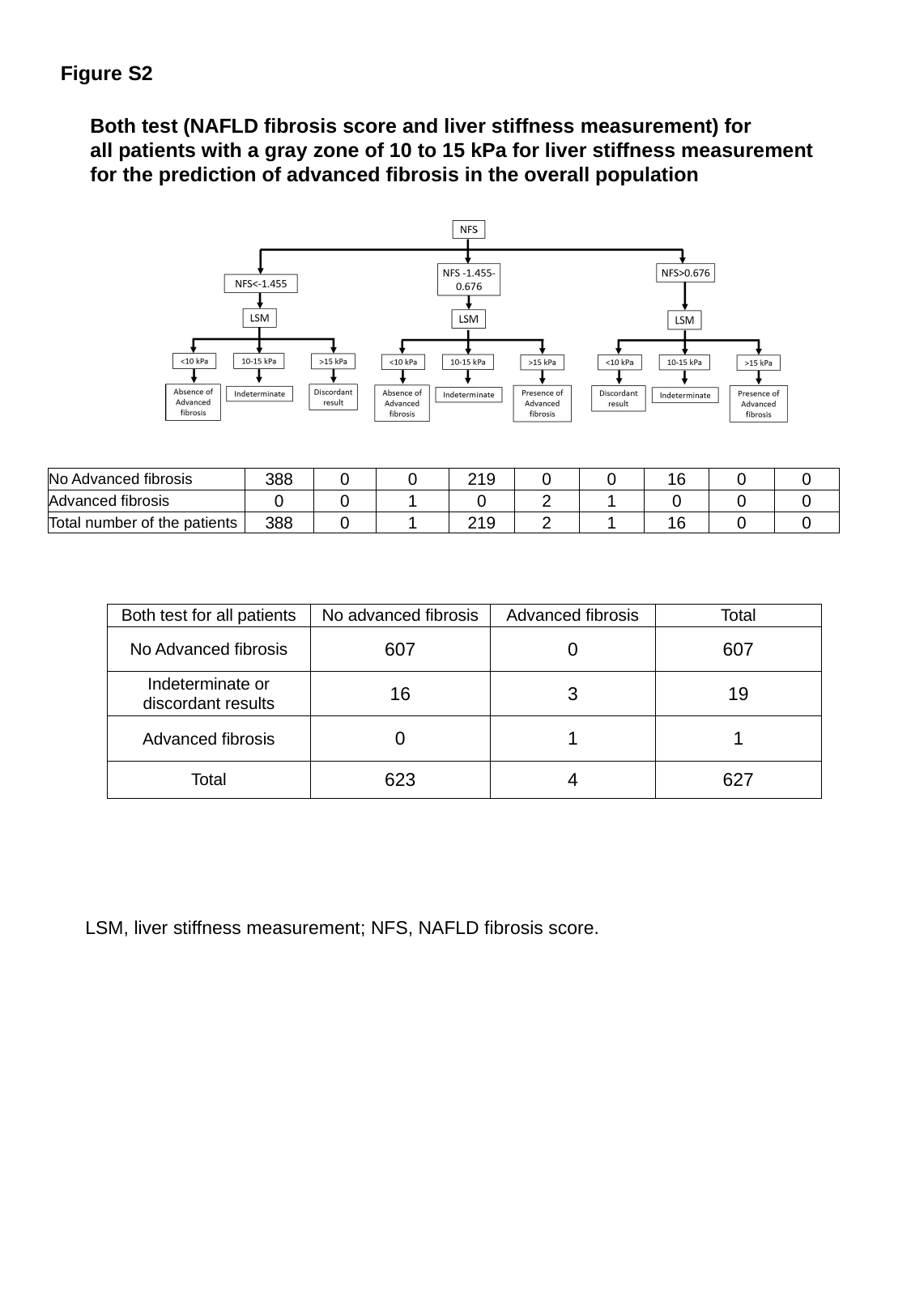

Figure S2
Both test (NAFLD ﬁbrosis score and liver stiffness measurement) for
all patients with a gray zone of 10 to 15 kPa for liver stiffness measurement
for the prediction of advanced ﬁbrosis in the overall population
| No Advanced fibrosis | 388 | 0 | 0 | 219 | 0 | 0 | 16 | 0 | 0 |
| --- | --- | --- | --- | --- | --- | --- | --- | --- | --- |
| Advanced fibrosis | 0 | 0 | 1 | 0 | 2 | 1 | 0 | 0 | 0 |
| Total number of the patients | 388 | 0 | 1 | 219 | 2 | 1 | 16 | 0 | 0 |
| Both test for all patients | No advanced fibrosis | Advanced fibrosis | Total |
| --- | --- | --- | --- |
| No Advanced fibrosis | 607 | 0 | 607 |
| Indeterminate or discordant results | 16 | 3 | 19 |
| Advanced fibrosis | 0 | 1 | 1 |
| Total | 623 | 4 | 627 |
LSM, liver stiffness measurement; NFS, NAFLD fibrosis score.

## Slide 3
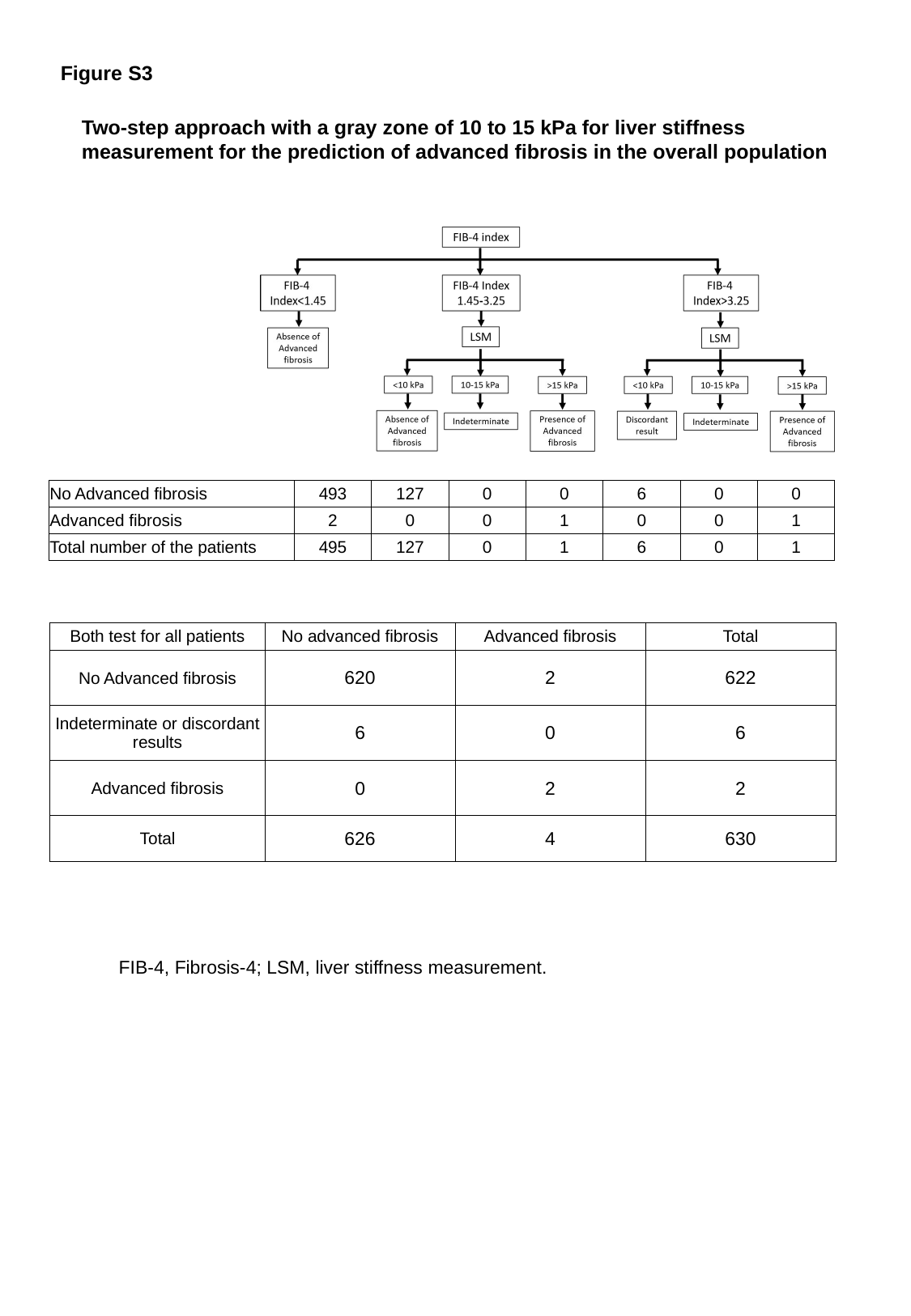

Figure S3
Two-step approach with a gray zone of 10 to 15 kPa for liver stiffness
measurement for the prediction of advanced ﬁbrosis in the overall population
| No Advanced fibrosis | 493 | 127 | 0 | 0 | 6 | 0 | 0 |
| --- | --- | --- | --- | --- | --- | --- | --- |
| Advanced fibrosis | 2 | 0 | 0 | 1 | 0 | 0 | 1 |
| Total number of the patients | 495 | 127 | 0 | 1 | 6 | 0 | 1 |
| Both test for all patients | No advanced fibrosis | Advanced fibrosis | Total |
| --- | --- | --- | --- |
| No Advanced fibrosis | 620 | 2 | 622 |
| Indeterminate or discordant results | 6 | 0 | 6 |
| Advanced fibrosis | 0 | 2 | 2 |
| Total | 626 | 4 | 630 |
FIB-4, Fibrosis-4; LSM, liver stiffness measurement.

## Slide 4
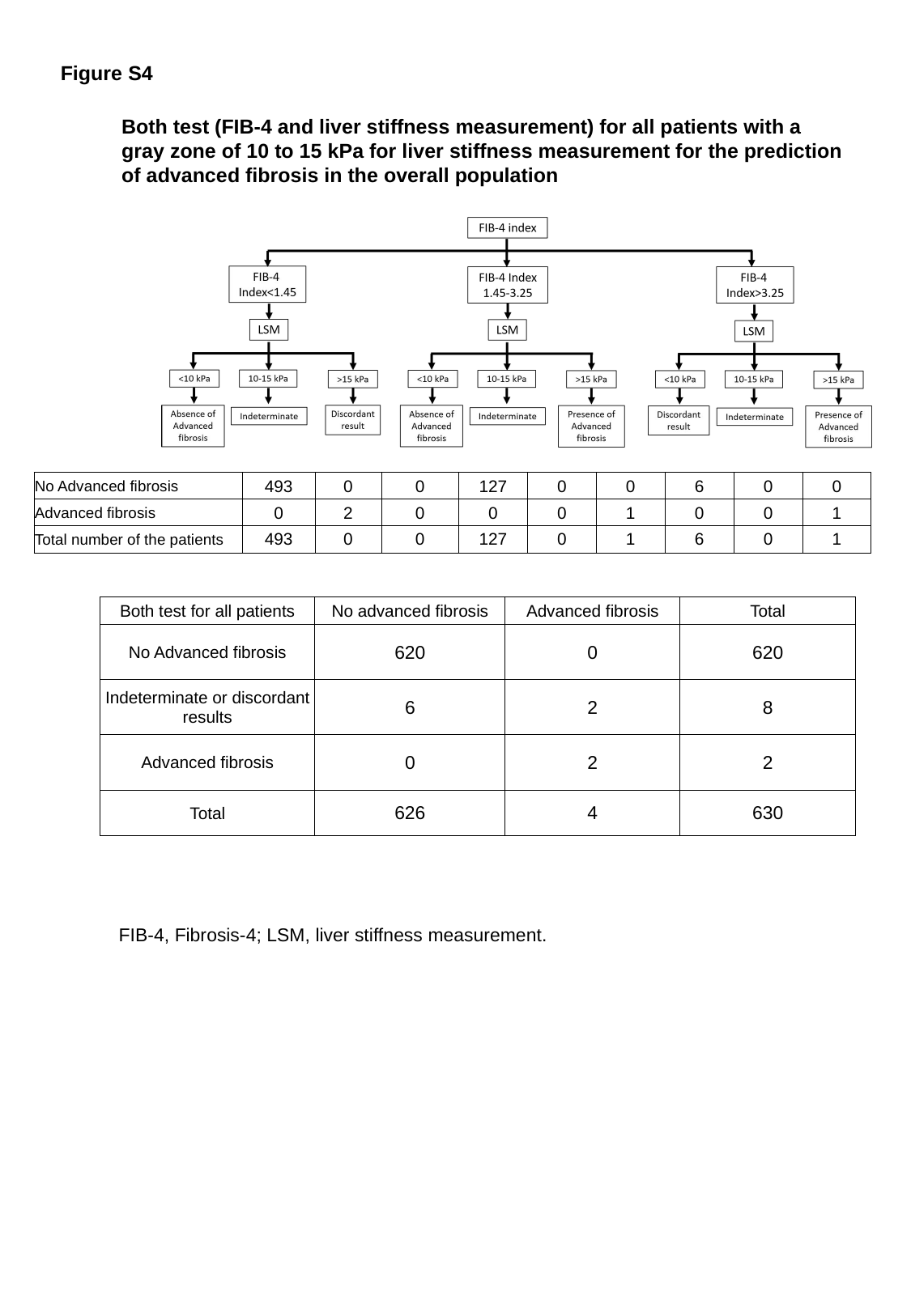

Figure S4
Both test (FIB-4 and liver stiffness measurement) for all patients with a
gray zone of 10 to 15 kPa for liver stiffness measurement for the prediction
of advanced ﬁbrosis in the overall population
| No Advanced fibrosis | 493 | 0 | 0 | 127 | 0 | 0 | 6 | 0 | 0 |
| --- | --- | --- | --- | --- | --- | --- | --- | --- | --- |
| Advanced fibrosis | 0 | 2 | 0 | 0 | 0 | 1 | 0 | 0 | 1 |
| Total number of the patients | 493 | 0 | 0 | 127 | 0 | 1 | 6 | 0 | 1 |
| Both test for all patients | No advanced fibrosis | Advanced fibrosis | Total |
| --- | --- | --- | --- |
| No Advanced fibrosis | 620 | 0 | 620 |
| Indeterminate or discordant results | 6 | 2 | 8 |
| Advanced fibrosis | 0 | 2 | 2 |
| Total | 626 | 4 | 630 |
FIB-4, Fibrosis-4; LSM, liver stiffness measurement.

## Slide 5
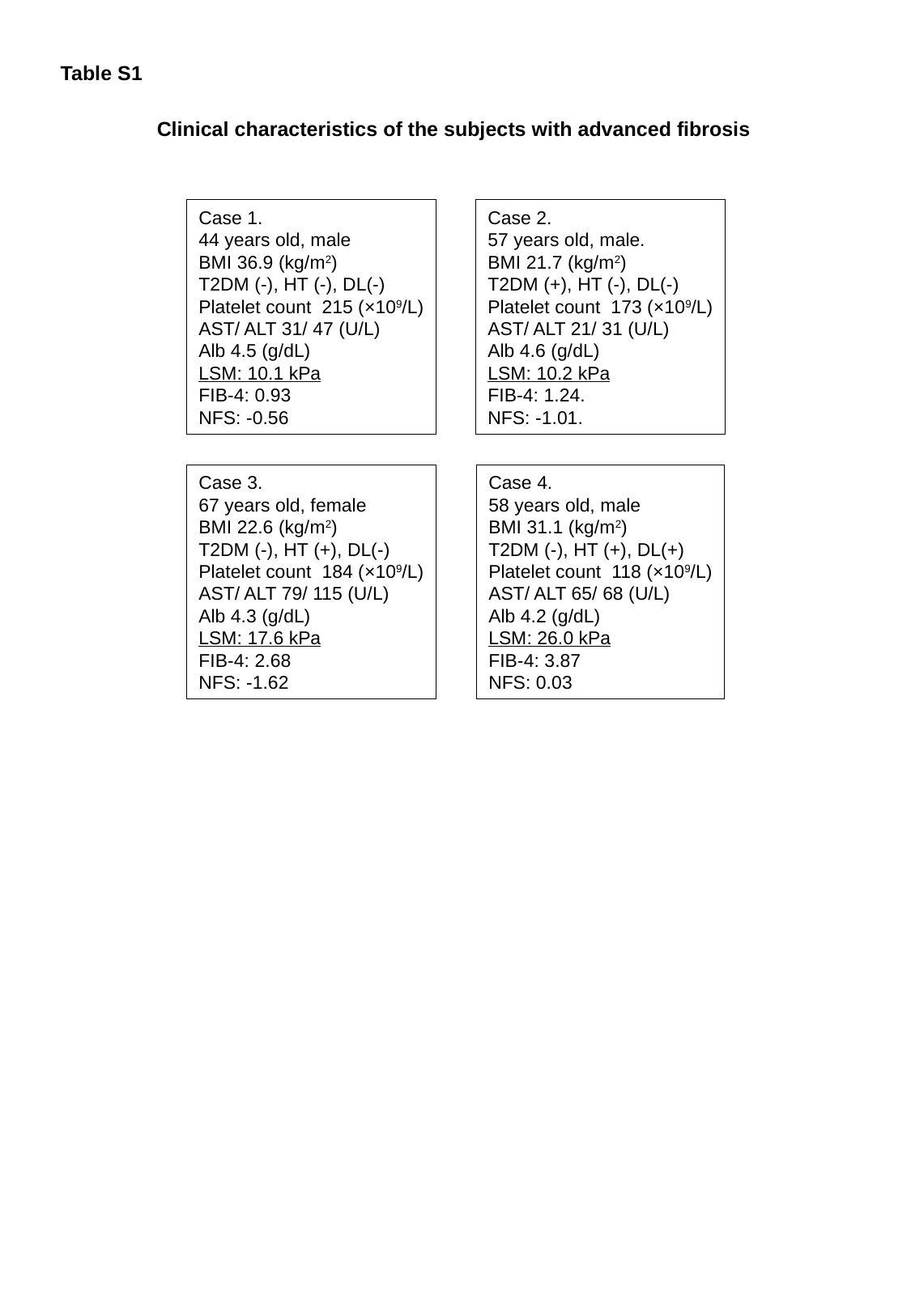

Table S1
Clinical characteristics of the subjects with advanced fibrosis
Case 1.
44 years old, male
BMI 36.9 (kg/m2)
T2DM (-), HT (-), DL(-)
Platelet count 215 (×109/L)
AST/ ALT 31/ 47 (U/L)
Alb 4.5 (g/dL)
LSM: 10.1 kPa
FIB-4: 0.93
NFS: -0.56
Case 2.
57 years old, male.
BMI 21.7 (kg/m2)
T2DM (+), HT (-), DL(-)
Platelet count 173 (×109/L)
AST/ ALT 21/ 31 (U/L)
Alb 4.6 (g/dL)
LSM: 10.2 kPa
FIB-4: 1.24.
NFS: -1.01.
Case 3.
67 years old, female
BMI 22.6 (kg/m2)
T2DM (-), HT (+), DL(-)
Platelet count 184 (×109/L)
AST/ ALT 79/ 115 (U/L)
Alb 4.3 (g/dL)
LSM: 17.6 kPa
FIB-4: 2.68
NFS: -1.62
Case 4.
58 years old, male
BMI 31.1 (kg/m2)
T2DM (-), HT (+), DL(+)
Platelet count 118 (×109/L)
AST/ ALT 65/ 68 (U/L)
Alb 4.2 (g/dL)
LSM: 26.0 kPa
FIB-4: 3.87
NFS: 0.03

## Slide 6
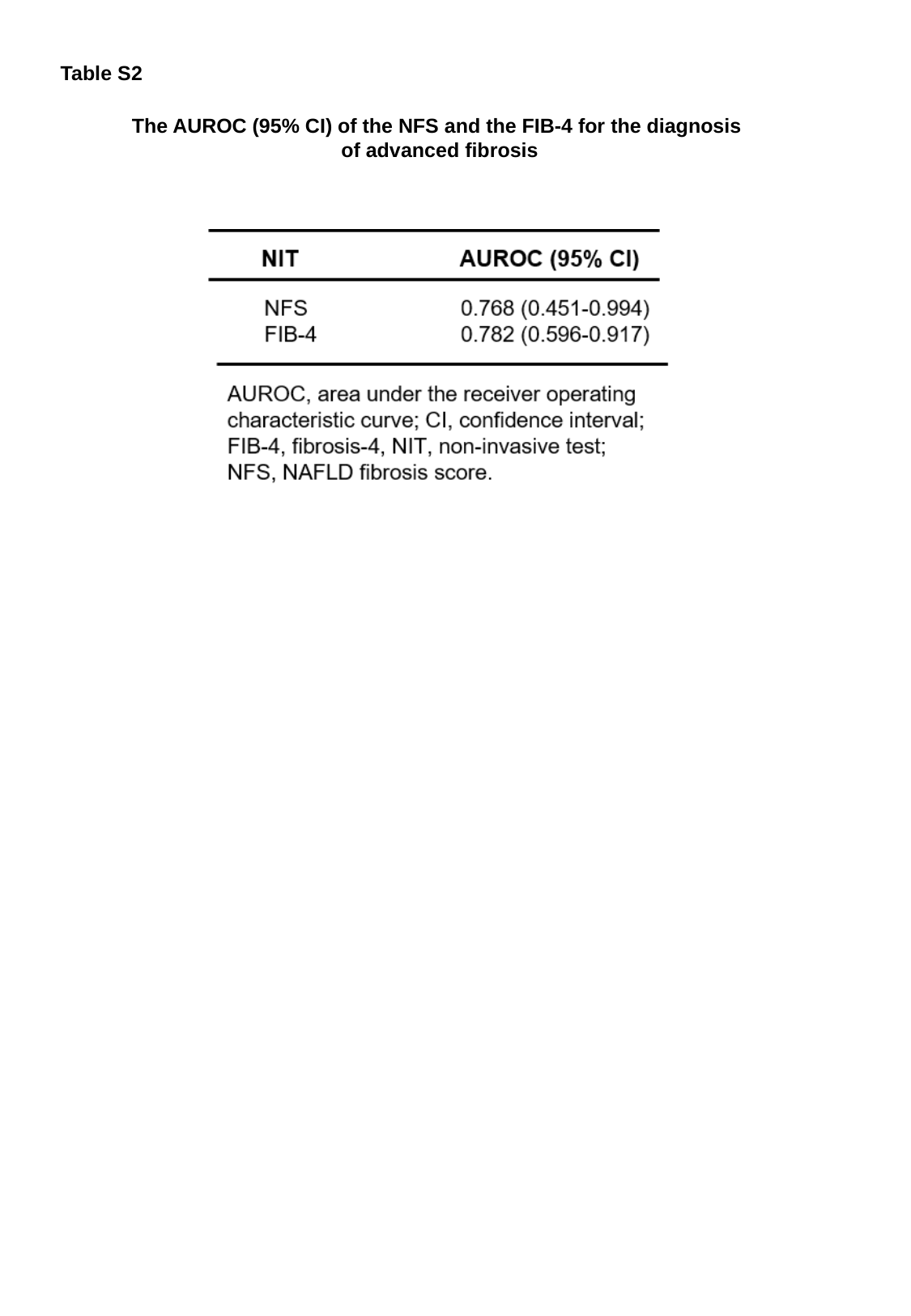

Table S2
The AUROC (95% CI) of the NFS and the FIB-4 for the diagnosis
of advanced fibrosis
